# The impact of macrosomia on cardiometabolic health in preteens: findings from the ROLO longitudinal birth cohort study

**DOI:** 10.1186/s12986-023-00759-8

**Published:** 2023-09-04

**Authors:** Sophie Callanan, Sarah Louise Killeen, Anna Delahunt, Nessa Cooney, Rosemary Cushion, Malachi J. McKenna, Rachel K. Crowley, Patrick J. Twomey, Mark T. Kilbane, Ciara M. McDonnell, Catherine M. Phillips, Declan Cody, Fionnuala M. McAuliffe

**Affiliations:** 1 UCD Perinatal Research Centre, School of Medicine, University College Dublin, The National Maternity Hospital, Dublin, Ireland; 2 Department of Endocrinology, St Vincent’s University Hospital, Dublin, Ireland; 3 Department of Clinical Chemistry, St Vincent’s University Hospital, Dublin, Ireland; 4 Department of Paediatric Endocrinology and Diabetes, Children’s Health Ireland, Temple Street and Tallaght, Dublin, Ireland; 5 School of Public Health, Physiotherapy and Sports Science, University College Dublin, Dublin, Ireland; 6 Department of Diabetes and Endocrinology, Children’s Health Ireland, Crumlin, Dublin, Ireland

**Keywords:** Birthweight, Macrosomia, ROLO, Cardiometabolic health, Birth cohort, Childhood obesity, Preteen

## Abstract

**Background:**

Macrosomia (birthweight ≥ 4 kg or ≥ 4.5 kg) is strongly associated with a predisposition to childhood obesity, which in turn is linked with adverse cardiometabolic health. Despite this, there is a lack of longitudinal investigation on the impact of high birthweight on cardiometabolic outcomes in youth. The preteen period represents an important window of opportunity to further explore this link, to potentially prevent cardiometabolic profiles worsening during puberty.

**Methods:**

This is a secondary analysis of 9–11-year-olds (n = 405) born to mothers in the ROLO longitudinal birth cohort study, who previously delivered an infant with macrosomia. Preteens were dichotomised into those born with and without macrosomia, using two common cut-off criteria (birthweight ≥ 4 kg (n = 208) and < 4 kg; ≥ 4.5 kg (n = 65) and < 4.5 kg). Cardiometabolic health was assessed using anthropometry, dual-energy x-ray absorptiometry, blood pressure, heart rate, cardiorespiratory endurance (20-m shuttle run test), and non-fasting serum biomarkers for a subgroup (n = 213). Statistical comparisons between the two groups were explored using independent *t*-tests, Mann–Whitney U tests, and Chi-square tests. Crude and adjusted linear regression models investigated associations between macrosomia and preteen cardiometabolic outcomes.

**Results:**

In total, 29.3% (n = 119) of preteens had overweight/obesity based on their BMI z-score. Preteens born ≥ 4 kg had lower median (IQR) C3 concentrations (1.38 (1.22, 1.52) g/L vs. 1.4 (1.26, 1.6) g/L, *p* = 0.043) and lower median (IQR) ICAM-1 concentrations (345.39 (290.34, 394.91) ng/mL vs. 387.44 (312.91, 441.83) ng/mL, *p* = 0.040), than those born < 4 kg. Those born ≥ 4.5 kg had higher mean (SD) BMI z-scores (0.71 (0.99) vs. 0.36 (1.09), *p* = 0.016), and higher median (IQR) lean mass (24.76 (23.28, 28.51) kg vs. 23.87 (21.9, 26.79) kg, *p* = 0.021), than those born < 4.5 kg. Adjusted linear regression analyses revealed birthweight ≥ 4 kg was negatively associated with C3 concentration (g/L) (B = − 0.095, 95% CI = − 0.162, − 0.029, *p* = 0.005) and birthweight ≥ 4.5 kg was positively associated with weight z-score (B = 0.325, 95% CI = 0.018, 0.633, *p* = 0.038), height z-score (B = 0.391, 95% CI = 0.079, 0.703, *p* = 0.014), lean mass (kg) (B = 1.353, 95% CI = 0.264, 2.442, *p* = 0.015) and cardiorespiratory endurance (B = 0.407, 95% CI = 0.006, 0.808, *p* = 0.047).

**Conclusion:**

This study found no strong evidence to suggest that macrosomia is associated with adverse preteen cardiometabolic health. Macrosomia alone may not be a long-term cardiometabolic risk factor.

*Trial registration* ISRCTN54392969 registered at www.isrctn.com.

**Supplementary Information:**

The online version contains supplementary material available at 10.1186/s12986-023-00759-8.

## Introduction

Cardiometabolic diseases represent a major cause of death in adults and the greatest financial burden in healthcare globally [1]. Early signs of cardiometabolic dysfunction can appear in the first decade of life [2, 3]. High rates of overweight and obesity are associated with increasing prevalence of chronic inflammation, immune dysregulation, and cardiovascular comorbidities in school-aged youth [4, 5]. The pubertal transition is linked with significant progression of cardiometabolic risk factors that become more challenging to reverse [6].

The developmental origins of health and disease paradigm supports a link between birthweight extremities, particularly low birthweight and long-term health [7]. High birthweight, or macrosomia, represents a challenging perinatal issue with lasting health implications [8]. The definition of macrosomia varies in the literature, usually referred to as a birthweight ≥ 4 kg or ≥ 4.5 kg [8]. Fetal overnutrition promotes hyperglycaemia and hyperinsulinemia, leading to extra fat accumulation that persists postnatally [9]. Thus, high birthweight has been highlighted as a reliable predictor of obesity in childhood and adolescence [10–13]. Several studies also support this phenomenon by linking high birthweight to excess fat mass in youth [14–16].

Observations in adults with high birthweight report increased blood pressure, triglycerides, and altered glucose metabolism [17, 18]. It is unclear if adverse cardiometabolic manifestations of high birthweight become apparent earlier in life. Few retrospective observations in youth include positive correlations between birthweight with blood pressure, unfavourable lipids, and poor glycaemic control [19–22]. The availability of robust evidence is further weakened by the ambiguity of results from few birth cohort studies that have examined high birthweight in relation to cardiometabolic disease risk [2, 15, 23–25].

To address potential inaccuracies arising from cardiometabolic assessment in early childhood, international clinical guidelines endorse preteen ages (9–11 years) as a more stable time point for cardiometabolic screening in both sexes as most will not have commenced puberty [3]. High birthweight individuals are more likely to deliver high birthweight offspring and this harmful pattern may contribute to an ongoing cycle of adverse maternal and offspring health [7, 9]. Thus, a case can be made for further longitudinal investigation of cardiometabolic health determinants in preteens, to enable greater impact for early intervention strategies in the prevention of later disease.

The aim of this study is to examine associations between macrosomia and cardiometabolic health at 9–11 years of age in preteens born to mothers in the ROLO (Randomised cOntrol trial of a LOw glycaemic index diet in pregnancy to prevent macrosomia) longitudinal birth cohort, who previously delivered an infant with macrosomia. We hypothesise that macrosomia may increase cardiometabolic risk in the preteen period, compared to preteens born without macrosomia.

## Methods

### The ROLO longitudinal birth cohort

The ROLO study was a randomised control trial of a low glycaemic diet in pregnancy to prevent the recurrence of macrosomia in secundigravida women [26]. The study was conducted in the National Maternity Hospital, Dublin, Ireland (2007–2011) and ethical approval was obtained from the Ethics Committee of the National Maternity Hospital (GEN/279/12). Detailed description of the methodology and results of the ROLO pregnancy study have been published previously [26]. The intervention involved the delivery of low glycaemic index dietary advice by a research dietitian, while those randomised to the control group received routine antenatal care which did not include any formal dietary advice. The primary outcome was birthweight, and no differences were noted in birthweight, though infants from the intervention group showed slightly lower thigh skinfolds [27]. The 759 mothers and children born into the pregnancy study have since been followed-up at multiple timepoints, with the most recent follow-up period taking place at 9–11 years postpartum. Written and informed consent was obtained from all eligible individuals prior to study participation.

### The ROLO preteen follow-up

Participants born into the ROLO pregnancy study who had available data on birthweight and attended a ROLO Preteen follow-up appointment between 9.0 and 11.9 years of age were eligible for inclusion. Once the study child became eligible for follow-up, mothers were phoned or emailed by the research team and invited to attend a follow-up visit at the Institute for Sport and Health in University College Dublin. At the 9–11 year follow-up, preteens in which a body mass index (BMI) z-score had been obtained were included, leading to a total sample of 405 preteens. This parameter was chosen to set the sample size to maximise power, and complete data for other variables varied depending on data collection at the time of follow-up.

### Exposure

The primary exposure of interest for this analysis was macrosomia, using two common definitions. Preteens were dichotomised as those who were born ≥ 4 kg (n = 208) and those born < 4 kg (n = 197). Subsequently, preteens were dichotomised into those born ≥ 4.5 kg (n = 65) and those born < 4.5 kg (n = 340) to further tease apart potential differences. The secondary exposure of interest for this analysis was birthweight centile, using the cut-off for “large-for-gestational-age”. At delivery, the Gestation Network’s Bulk Calculator version 6.2.3 UK was used to calculate birthweight centiles [28]. Preteens were dichotomised as those who were born with a birthweight ≥ 90th centile (n = 159) and those born < 90th centile (n = 245).

### Outcomes

#### Anthropometry and body composition

At 9–11 years of age, weight and height were assessed by a trained research nutritionist/dietitian with participants dressed in light clothing and shoes removed. Weight was measured using the calibrated SECA model flat, mobile weighing scales, to the nearest 0.1 kg (SECA GmbH & co. Kg. Germany). Height was measured using the SECA 123 portable stadiometer, to the nearest 0.1 cm (SECA GmbH & co. Kg. Germany). BMI was calculated as kg/m^2^ and values were converted to z-scores in line with the 1990 UK reference data [29, 30]. BMI z-scores were categorised according to World Health Organization cut-offs; “underweight” (BMI z-score < − 2.0); “healthy weight” (BMI z-score > − 2.0 and ≤ 1.0); “overweight” (BMI z-score > 1.0); “obesity” (BMI z-score > 2.0) [31]. Circumferences were measured at the mid-upper arm and waist (at the point of the umbilicus), using the SECA 201 ergonomic circumference measuring tape, to the nearest 0.1 cm (SECA GmbH & co. Kg. Germany). Skinfold thickness were assessed at three sites (biceps, triceps, and subscapular) using a Holtain Tanner/Whitehouse skinfold calipers, to the nearest 0.2 mm (Holtain Ltd, Crymych, UK). The sum of three skinfolds and subscapular/triceps ratio were calculated as proxy measures of adiposity. Dual-energy x-ray absorptiometry (DXA) was performed for 348 participants using the Lunar iDXA™ scanner (GE Healthcare, Madison WI) with enCORE™ v.18.0 software. Measurements included total lean mass (kg) and percentage body fat.

#### Cardiovascular health

Cardiovascular health parameters were assessed using blood pressure and heart rate at rest. A validated electronic sphygmomanometer (Omron M6 HEM-7211-E8(V)) was used to assess systolic blood pressure (SBP), diastolic blood pressure (DBP) and resting heart rate. Values were measured twice with a 1-min interval between each reading and an average was determined. If the difference between the first two readings was > 10%, a third measurement was taken and the mean of three measurements was used. SBP and DBP percentiles were calculated for each participant according to sex, age, and height-specific reference data [32].

#### Cardiorespiratory endurance

Preteens completed the validated 20-m shuttle run test which followed previously described protocols [33]. A pre-recorded sound signal was emitted that had a starting speed of 8.5 km h^−1^ and increased by 0.5 km h^−1^ every 60 s. Participants ran in consecutive stages back and forth to measured end-points on a linear 20 m indoor track. Preteens were instructed to reach the endpoints before the sound was emitted and the test ended once they could no longer follow the pace of the bleep by failing to reach the end lines on two consecutive occasions. The last stage number was used to predict cardiorespiratory endurance from the score obtained.

#### Cardiometabolic biomarkers

Non-fasting blood samples were obtained from 213 preteens. We analysed several traditional and non-traditional biomarkers that have been previously associated with adverse cardiometabolic functioning in youth [4, 34, 35]. Serum concentrations of glucose, insulin, total cholesterol, triglycerides, high density lipoprotein cholesterol (HDL-C), c-reactive protein, and C3 complement protein (C3) were all analysed on the Cobas c701/702 module of the Roche Cobas 8000 analyser (Roche Diagnostics GmbH, Penzburg, Germany). Homeostatic Model Assessment for Insulin Resistance (HOMA-IR) index was calculated using the standard formula (glucose (mmol/L) x insulin (mIU/L) / 22.5) [10]. Low density lipoprotein cholesterol (LDL-C) was calculated using the Friedewald equation [36]. Serum concentrations of intracellular adhesion molecule 1 (ICAM-1), tumour necrosis factor alpha (TNF-α), growth differentiation factor-15 (GDF-15), soluble cluster of differentiation factor 163 (sCD163), leptin, interleukin-6 (IL-6), and interleukin-17A (IL-17A) were all quantified using the benchtop automated ELISA platform ProteinSimple Ella™ (Bio-Techne) following the manufacturer’s instructions. For all biomarkers, outliers more than 5 standard deviations from the mean were excluded.

### Covariates

**Fig. 1:**
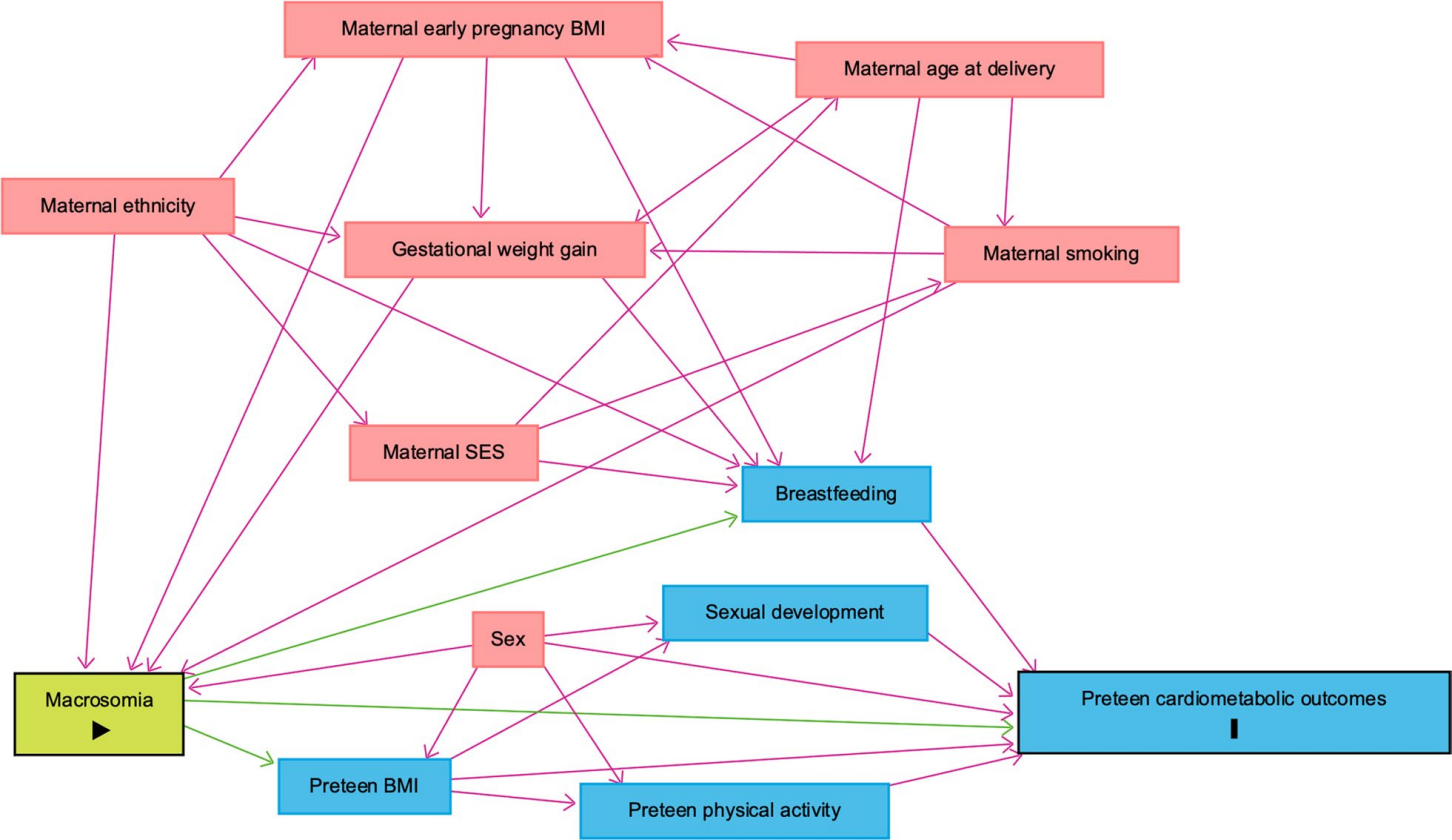
Directed acyclic graph of preteen cardiometabolic outcomes. Abbreviations: *BMI* Body mass index, *SES* Socio-economic status

The a priori selection of potential confounding factors to include in multiple linear regression models was informed by the literature [37–40] and by a directed acyclic graph (DAG) (Fig. 1). Study group allocation and age at the time of the 9–11 year follow-up were controlled for to account for differences within the sample population. The ROLO pregnancy study collected data on maternal characteristics which have been described previously [41, 42]. Information included ethnicity, smoking status in pregnancy, early pregnancy BMI (obtained at the first antenatal appointment), gestational weight gain (adherence to 2009 Institute of Medicine guidelines), age at delivery, and socio-economic status using the Pobal Hasse and Pratschke Deprivation Index (HP index). Information on breastfeeding exposure and duration was obtained at 6 months and 2, 5, and 9–11 year postnatal follow up visits. Mothers reported estimates of their preteens’ sexual development using standardised Tanner staging figures (from Stage 1 to 5) [43]. At 9–11 years, self-reported physical activity was assessed using the validated Physical Activity Questionnaire for Older Children (PAQ-C), with calculated mean scores ranging from 1 (low physical activity levels) to 5 (high physical activity levels) [44].

### Statistical analysis

All statistical analyses were carried out using the IBM Statistical Package for the Social Sciences Statistics for Mac Version 27.0 (Macintosh, Armonk, NY). Normality was assessed for all continuous variables using the Kolmogorov–Smirnov tests and visual inspection of simple histograms. Normally distributed variables were reported as mean (standard deviation (SD)), non-normally distributed variables were reported as median (interquartile range 25th–75th percentile (IQR)), and categorical variables were reported as n (%). Non-normal data was log transformed prior to analysis. Differences between those born with macrosomia and those without were investigated using independent *t*-tests, Mann–Whitney U tests, and Chi-square tests as appropriate.

Crude and adjusted multiple linear regression models were created to examine associations between birthweight ≥ 4 kg (yes/no); birthweight ≥ 4.5 kg (yes/no); birthweight ≥ 90th centile (yes/no); and cardiometabolic outcomes at 9–11 years of age using a forced entry approach. Model 1 presents crude results; Model 2 was adjusted for confounders informed by the DAG (maternal ethnicity (White Irish, yes/no), HP index, maternal age at delivery (years), smoking in pregnancy (yes/no), early pregnancy BMI (kg/m^2^), adherence to gestational weight gain guidelines (inadequate/adequate/excessive), and child sex (male, yes/no)), along with study group allocation (intervention, yes/no) and age of the preteen at the time of follow-up (years); Model 3 was additionally adjusted for breastfeeding exposure (never breastfed/breastfed < 2 months/breastfed ≥ 2 and < 4 months/breastfed ≥ 4 months) for all outcomes and preteen BMI (kg/m^2^) for outcomes of cardiovascular health, fitness, and laboratory biomarkers only.

Sensitivity analyses were performed to examine the additional effect of preteen lifestyle and biological factors on the outcomes of interest based on previous literature [45, 46]. Linear regression models were repeated and further adjusted for sexual development (Tanner stage 1, yes/no) and preteen physical activity level (PAQ-C score) for all outcomes. All analyses were performed with pairwise deletion of missing data. A *p*-value < 0.05 was considered statistically significant for all analyses. Correction for multiple testing was not applied due to the exploratory design of this analysis [47].

## Results

### Maternal and child characteristics in the ROLO longitudinal birth cohort

A total of 405 preteens were included in this analysis and the cohort were evenly distributed by sex; 50.1% (n = 203) were male. The mean (SD) birthweight was 4.03 (0.46) kg and ranged from 2.66 to 5.35 kg. The median (IQR 25th–75th percentile) birthweight centile was 86.32 (72.24, 95.68) and 39.4% (n = 159) were born ≥ 90th centile. 51.4% (n = 208) of preteens were born with macrosomia and 16% (n = 65) were born ≥ 4.5 kg. The median (IQR 25th–75th percentile) age of follow-up was 9.83 (9.24, 10.27) years. Further details of cohort characteristics are displayed in Table 1. There were no significant differences in baseline maternal and infant characteristics between the follow-up cohort at 9–11 years and the original ROLO pregnancy cohort (Additional file 1: Table 1).

**Table 1:** Maternal, infant, and preteen characteristics of the ROLO longitudinal birth cohort study

	Total (n = 405)		
	N	Mean/Median/n	SD/(IQR)/%
*Maternal characteristics*			
Age at delivery (years)	404	33.17	(30.5, 35.47)
Early pregnancy BMI (kg/m^2^)	401	25.46	(23.3, 27.96)
HP index	405	7.5	(1.1, 12.5)
Gestational weight gain			
Inadequate, n (%)	333	52	15.6
Adequate, n (%)	333	127	38.1
Excessive, n (%)	333	154	46.2
Ethnicity (White Irish), n (%)	405	372	91.9
Smoking in pregnancy, n (%)	405	12	3.2
RCT group (intervention), n (%)	405	203	50.1
*Infant characteristics*			
Child sex (male), n (%)	405	203	50.1
Birthweight (kg)	405	4.03	0.46
Birthweight centile	404	86.32	(72.24, 95.68)
Gestational age at delivery (days)	404	283.0	(277.0, 288.0)
Birthweight ≥ 4 kg, n (%)	405	208	51.4
Birthweight ≥ 4.5 kg, n (%)	405	65	16.0
Birthweight ≥ 90th centile, n (%)	404	159	39.4
Breastfeeding exposure and duration			
Never breastfed, n (%)	360	133	36.9
Breastfed < 2 months, n (%)	360	46	12.8
Breastfed ≥ 2 and < 4 months, n (%)	360	39	10.8
Breastfed ≥ 4 months, n (%)	360	142	39.4
*Preteen characteristics*			
Age at follow-up (years)	405	9.83	(9.24, 10.27)
Pubic hair distribution (Tanner stage I), n (%)	320	283	88.4
Breast development (Tanner stage I), n (%)	163	128	78.5
Physical activity level (PAQ-C score)	370	2.48	0.66

Results presented as mean (SD) for normally distributed variables and median (IQR 25th–75th percentile) for non-normally distributed variables. N = total population with available data; n = frequency. *ROLO* Randomised cOntrol trial of LOw glycaemic index diet in pregnancy versus no dietary intervention to prevent recurrence of macrosomia, *SD* Standard deviation, *IQR* Interquartile range, *BMI* Body mass index, *HP* Hasse and Pratschke Deprivation index, *RCT* Randomised control trial, *PAQ-C* Physical Activity Questionnaire for Older Children

### Anthropometry and body composition at the 9–11 year follow-up

At 9–11 years of age, the median (IQR 25th–75th percentile) BMI was 17.3 (15.85, 19.55) kg/m^2^. Based on BMI z-score classification, 21.2% (n = 86) of preteens had overweight and 8.1% (n = 33) had obesity (Table 2). No significant differences were found in any of the anthropometric measurements between preteens born ≥ 4 kg and < 4 kg (Table 3). When dichotomised into preteens born ≥ 4.5 kg and < 4.5 kg, preteens born ≥ 4.5 kg had higher mean (SD) weight z-scores (0.88 (0.88) vs. 0.48 (0.99), *p* = 0.003), higher mean (SD) height z-scores (0.81 (0.86) vs. 0.46 (0.96), *p* = 0.007), higher mean (SD) BMI z-scores (0.71 (0.99) vs. 0.36 (1.09), *p* = 0.016), and higher median (IQR 25th–75th percentile) lean mass (24.76 (23.28, 28.51) kg vs. 23.87 (21.9, 26.79) kg, *p* = 0.021), compared to preteens born < 4.5 kg (Table 4). Preteens born with a birthweight ≥ 90th centile had higher mean (SD) weight z-scores (0.7 (0.92) vs. 0.44 (1.01), *p* = 0.010), higher median (IQR 25th–75th percentile) height (141.4 (136.8, 145.8) cm vs. 139.1 (134.4, 146.6) cm, *p* = 0.028), higher mean (SD) height z-scores (0.73 (0.91) vs. 0.38 (0.96), *p* < 0.001), lower median (IQR 25th–75th percentile) subscapular/triceps ratio (0.57 (0.47, 0.67) vs. 0.6 (0.51, 0.75), *p* = 0.004), and higher median (IQR 25th–75th percentile) lean mass (24.56 (22.95, 27.23) kg vs. 23.7 (21.75, 26.89) kg, *p* = 0.015), than those born < 90th centile (Additional file 2: Table 2).

**Table 2:** Descriptive cardiometabolic outcomes of preteens

	Total (n = 405)		
	N	Mean/Median/n	SD/(IQR)/%
*Anthropometry and body composition*			
Weight (kg)	405	33.8	(30.4, 39.9)
Weight z-score	405	0.55	0.98
Height (cm)	405	140.1	(135.5, 146.3)
Height z-score	405	0.52	0.96
BMI (kg/m^2^)	405	17.3	(15.85, 19.55)
BMI z-score	405	0.41	1.08
Overweight, n (%)	405	86	21.2
Obesity, n (%)	405	33	8.1
MUAC (cm)	401	21.0	(19.1, 23.2)
WC (cm)	405	62.3	(58.7, 68.0)
Sum of skinfolds (mm)	366	28.28	(21.58, 36.84)
Subscapular/triceps ratio	375	0.59	(0.49, 0.72)
Lean mass (kg)	348	24.14	(22.02, 26.91)
Body fat (%)	348	26.3	(20.9, 31.97)
*Cardiovascular health and cardiorespiratory endurance*			
SBP (mmHg)	380	111.0	(104.0, 117.0)
SBP percentile	380	88.0	(68.0, 96.0)
DBP (mmHg)	380	67.0	(61.75, 72.0)
DBP percentile	380	73.5	(53.0, 87.0)
Resting heart rate (bpm)	377	79.07	12.66
20-M SRT score	378	3.55	(3.1, 4.52)
*Cardiometabolic biomarkers*			
HOMA-IR	210	2.46	(1.38, 5.11)
TC (mmol/L)	212	4.15	0.69
Triglycerides (mmol/L)	213	0.88	(0.69, 1.29)
LDL-C (mmol/L)	212	2.41	0.55
HDL-C (mmol/L)	213	1.24	(0.96, 1.5)
C-reactive protein (mg/L)	202	0.25	(0.12, 0.46)
C3 complement (g/L)	213	1.39	(1.23, 1.57)
ICAM-1 (ng/mL)	163	357.24	(297.6, 419.04)
TNF-α (pg/mL)	163	8.64	2.71
GDF-15 (ng/mL)	163	0.29	(0.24, 0.35)
sCD163 (ng/mL)	163	429.53	(338.42, 554.93)
Leptin (ng/mL)	162	4.53	(1.72, 8.81)
IL-6 (pg/mL)	162	0.74	(0.49, 1.09)
IL-17A (pg/mL)	163	1.27	(0.84, 2.13)

Results presented as mean (SD) for normally distributed variables, median (IQR 25th–75th percentile) for non-normally distributed variables, and n (%) for categorical variables. N = total population with available data; n = frequency. *ROLO* Randomised cOntrol trial of LOw glycaemic index diet in pregnancy versus no dietary intervention to prevent recurrence of macrosomia, *SD* Standard deviation, *IQR* Interquartile range, *BMI* Body mass index, *MUAC* Mid-upper arm circumference, *WC* Waist circumference, *SBP* Systolic blood pressure, *DBP* Diastolic blood pressure, *20-M* SRT 20-m shuttle run test, *HOMA-IR* Homeostatic Model Assessment for Insulin Resistance, *TC* Total cholesterol, *LDL-C* Low density lipoprotein cholesterol, *HDL-C* High density lipoprotein cholesterol, *ICAM-1* Intracellular adhesion molecule 1, *TNF-α* Tumour necrosis factor alpha, *GDF-15* Growth differentiation factor 15, *sCD163* Soluble cluster of differentiation factor 163, *IL* Interleukin

**Table 3:** Comparison of cardiometabolic health in 9–11-year-old preteens born ≥ 4 kg and < 4 kg

	Birthweight ≥ 4 kg			Birthweight < 4 kg			*p*
	N	Mean/Median/n	SD/(IQR)/%	N	Mean/Median/n	SD/(IQR)/%	
*Anthropometry and body composition*							
Weight (kg)	208	34.4	(30.45, 40.15)	197	33.6	(30.3, 39.6)	0.598
Weight z-score	208	0.63	0.94	197	0.45	1.01	0.064
Height (cm)	208	141.06	7.37	197	140.98	7.98	0.911
Height z-score	208	0.61	0.91	197	0.43	1.33	0.059
BMI (kg/m^2^)	208	17.38	(16.03, 19.48)	197	17.14	(15.62, 19.66)	0.409
BMI z-score	208	0.48	1.03	197	0.34	1.13	0.169
Overweight, n (%)	208	51	24.5	197	35	17.8	0.141
Obesity, n (%)	208	14	6.7	197	19	9.6	
MUAC (cm)	206	21.0	(19.1, 23.0)	196	21.0	(19.2, 23.6)	0.977
WC (cm)	208	62.35	(58.42, 67.42)	197	62.0	(58.8, 68.05)	0.752
Sum of skinfolds (mm)	192	27.06	(21.2, 36.5)	188	29.5	(22.3, 37.83)	0.167^a^
Subscapular/triceps ratio	189	0.58	(0.48, 0.7)	186	0.6	(0.51, 0.74)	0.098
Lean mass (kg)	178	24.4	(22.3, 27.14)	170	23.77	(21.87, 26.38)	0.063
Body fat (%)	178	25.3	(20.42, 31.9)	170	26.5	(21.8, 32.1)	0.218^a^
*Cardiovascular health and cardiorespiratory endurance*							
SBP percentile	194	86.5	(70.25, 96.0)	186	89.5	(66.75, 96.0)	0.747
DBP percentile	194	73.0	(50.0, 86.0)	186	74.0	(55.0, 88.0)	0.380
Resting heart rate (bpm)	192	79.42	12.25	185	78.7	13.1	0.579
20-M SRT score	193	3.7	(3.1, 5.05)	185	3.5	(3.1, 4.4)	0.165
*Cardiometabolic biomarkers*							
HOMA-IR	104	2.23	(1.18, 5.06)	106	2.63	(1.55, 5.15)	0.169^a^
TC (mmol/L)	104	4.09	0.72	108	4.22	0.66	0.171
Triglycerides (mmol/L)	105	0.83	(0.67, 1.16)	108	0.91	(0.69, 1.49)	0.243
LDL-C (mmol/L)	104	2.39	0.59	108	2.42	0.53	0.723
HDL-C (mmol/L)	105	1.22	0.38	108	1.26	0.37	0.426
C-reactive protein (mg/L)	98	0.23	(0.11, 0.42)	104	0.26	(0.13, 0.54)	0.335^a^
C3 complement (g/L)	105	1.38	(1.22, 1.52)	108	1.4	(1.26, 1.6)	0.043^a^
ICAM-1 (ng/mL)	79	345.39	(290.34, 394.91)	84	387.44	(312.91, 441.83)	0.040^a^
TNF-α (pg/mL)	79	8.37	2.75	84	8.9	2.67	0.220
GDF-15 (ng/mL)	79	0.29	(0.24, 0.33)	84	0.3	(0.25, 0.38)	0.214
sCD163 (ng/mL)	79	398.61	(332.86, 510.87)	84	452.41	(340.26, 574.09)	0.143^a^
Leptin (ng/mL)	79	4.38	(1.58, 9.03)	83	4.86	(1.76, 8.52)	0.672^a^
IL-6 (pg/mL)	78	0.7	(0.46, 1.11)	84	0.76	(0.51, 1.05)	0.315^a^
IL-17A (pg/mL)	79	1.18	(0.83, 1.84)	84	1.32	(0.94, 2.2)	0.177

Results presented as mean (SD) for normally distributed variables, median (IQR 25th–75th percentile) for non-normally distributed variables, and n (%) for categorical variables. N = total population with available data; n = frequency. ^a^log10 transformed data was used. *SD* Standard deviation; IQR Interquartile range, *BMI* Body mass index, *MUAC* Mid-upper arm circumference, *WC* Waist circumference, *SBP* Systolic blood pressure, *DBP* Diastolic blood pressure, *20-M SRT* 20-m shuttle run test, *HOMA-IR* Homeostatic Model Assessment for Insulin Resistance, *TC* Total cholesterol, *LDL-C* Low density lipoprotein cholesterol, *HDL-C* High density lipoprotein cholesterol, *ICAM-1* Intracellular adhesion molecule 1, *TNF-α* Tumour necrosis factor alpha, *GDF-15* Growth differentiation factor 15, *sCD163* Soluble cluster of differentiation factor 163, *IL* Interleukin. *P* values determined using independent *t*-tests for normally distributed variables; Mann–Whitney U tests for non-normally distributed variables; Chi square tests for categorical variables

**Table 4:** Comparison of cardiometabolic health in 9–11-year-old preteens born ≥ 4.5 kg and < 4.5 kg

	Birthweight ≥ 4.5 kg			Birthweight < 4.5 kg			*p*
	N	Mean/Median/n	SD/(IQR)/%	N	Mean/Median/n	SD/(IQR)/%	
*Anthropometry and body composition*							
Weight (kg)	65	35.6	(31.6, 40.5)	340	33.8	(30.0, 39.75)	0.111
Weight z-score	65	0.88	0.88	340	0.48	0.99	0.003
Height (cm)	65	142.05	6.82	340	140.82	7.81	0.237
Height z-score	65	0.81	0.86	340	0.46	0.96	0.007
BMI (kg/m^2^)	65	17.86	(16.36, 19.43)	340	17.12	(15.69, 19.64)	0.068
BMI z-score	65	0.71	0.99	340	0.36	1.09	0.016
Overweight, n (%)	65	18	27.7	340	68	20.0	0.464
Obesity, n (%)	65	6	9.2	340	27	7.9	
MUAC (cm)	65	21.1	(19.55, 23.5)	336	21.0	(19.1, 23.2)	0.447
WC (cm)	65	63.2	(59.7, 68.1)	340	62.0	(58.25, 68.0)	0.159
Sum of skinfolds (mm)	60	26.53	(21.51, 37.48)	306	28.43	(21.58, 36.48)	0.808
Subscapular/triceps ratio	61	0.58	(0.46, 0.69)	314	0.59	(0.5, 0.72)	0.157
Lean mass (kg)	57	24.76	(23.28, 28.51)	291	23.87	(21.9, 26.79)	0.021
Body fat (%)	57	25.3	(20.35, 31.85)	291	26.4	(20.9, 32.1)	0.604
Cardiovascular health and cardiorespiratory endurance							
SBP percentile	60	86.0	(72.25, 95.75)	320	88.5	(67.0, 96.0)	0.723
DBP percentile	60	64.5	(46.25, 81.75)	320	74.0	(54.0, 88.0)	0.038
Resting heart rate (bpm)	60	78.45	13.23	317	79.18	12.57	0.682
20-M SRT score	61	3.8	(3.1, 5.15)	317	3.5	(3.1, 4.5)	0.333
*Cardiometabolic biomarkers*							
HOMA-IR	35	2.48	(1.56, 6.07)	175	2.45	(1.36, 4.6)	0.413^a^
TC (mmol/L)	35	4.03	0.65	177	4.18	0.7	0.264
Triglycerides (mmol/L)	36	0.92	(0.68, 1.58)	177	0.88	(0.69, 1.24)	0.352
LDL-C (mmol/L)	35	2.26	0.53	177	2.44	0.56	0.082
HDL-C (mmol/L)	36	1.21	0.39	177	1.25	0.37	0.623
C-reactive protein (mg/L)	35	0.22	(0.11, 0.42)	167	0.25	(0.13, 0.49)	0.779^a^
C3 complement (g/L)	36	1.38	(1.2, 1.56)	177	1.39	(1.24, 1.58)	0.486^a^
ICAM-1 (ng/mL)	22	322.19	(291.97, 390.87)	141	358.2	(300.91, 426.82)	0.106
TNF-α (pg/mL)	22	8.53	2.09	141	8.66	2.81	0.842
GDF-15 (ng/mL)	22	0.29	(0.23, 0.33)	141	0.29	(0.25, 0.35)	0.436
sCD163 (ng/mL)	22	394.95	(344.01, 544.07)	141	430.66	(335.64, 556.24)	0.720^a^
Leptin (ng/mL)	22	2.52	(1.53, 10.38)	140	4.9	(1.76, 8.73)	0.625
IL-6 (pg/mL)	21	0.62	(0.44, 1.14)	141	0.75	(0.5, 1.07)	0.278^a^
IL-17A (pg/mL)	22	1.01	(0.76, 1.78)	141	1.3	(0.87, 2.13)	0.254

Results presented as mean (SD) for normally distributed variables, median (IQR 25th–75th percentile) for non-normally distributed variables, and n (%) for categorical variables. N = total population with available data; n = frequency. ^a^log10 transformed data was used. *SD* Standard deviation, *IQR* Interquartile range, *BMI* Body mass index, *MUAC* Mid-upper arm circumference, *WC* Waist circumference, *SBP* Systolic blood pressure, *DBP* Diastolic blood pressure, *20-M SRT* 20-m shuttle run test, *HOMA-IR* Homeostatic Model Assessment for Insulin Resistance, *TC* Total cholesterol, *LDL-C* Low density lipoprotein cholesterol, *HDL-C* High density lipoprotein cholesterol, *ICAM-1* Intracellular adhesion molecule 1, *TNF-α* Tumour necrosis factor alpha, *GDF-15* Growth differentiation factor 15, *sCD163* Soluble cluster of differentiation factor 163, *IL* Interleukin. *P* values determined using independent *t*-tests for normally distributed variables; Mann–Whitney U tests for non-normally distributed variables; Chi square tests for categorical variables

### Cardiovascular health and cardiorespiratory endurance at the 9–11 year follow-up

No significant differences were found in cardiovascular health parameters and cardiorespiratory endurance between preteens born ≥ 4 kg and < 4 kg (Table 3) and those born with a birthweight ≥ 90th and < 90th centile (Additional file 2: Table 2). When dichotomised into preteens born ≥ 4.5 kg and < 4.5 kg, preteens born ≥ 4.5 kg had lower median (IQR 25th–75th percentile) diastolic blood pressure percentile (64.5 (46.25, 81.75) vs. 74.0 (54.0, 88.0), *p* = 0.038), compared to those born < 4.5 kg (Table 4).

### Cardiometabolic biomarkers at the 9–11 year follow-up

Of 405 preteens included in this analysis, a non-fasting blood sample was obtained for 213 preteens (52.5%). Of these, 105 preteens (49.3%) were born ≥ 4 kg. Preteens born ≥ 4 kg had lower median (IQR 25th–75th percentile) C3 concentrations (1.38 (1.22, 1.52) g/L vs. 1.4 (1.26, 1.6) g/L, *p* = 0.043) and lower median (IQR 25th–75th percentile) ICAM-1 concentrations (345.39 (290.34, 394.91) ng/mL vs. 387.44 (312.91, 441.83) ng/mL, *p* = 0.040), compared to preteens born < 4 kg (Table 3). Preteens born with a birthweight ≥ 90th centile had lower median (IQR 25th–75th percentile) HOMA-IR index scores (2.0 (1.31, 4.45) vs. 3.06 (1.57, 5.34), *p* = 0.037) and lower median (IQR 25th–75th percentile) IL-17A concentrations (1.06 (0.73, 2.01) pg/mL vs. 1.33 (0.97, 2.18) pg/mL, *p* = 0.038), than those born < 90th centile (Additional file 2: Table 2).

### Regression between macrosomia and cardiometabolic outcomes at the 9–11 year follow-up

Multiple linear regression analyses examined associations between birthweight ≥ 4 kg; birthweight ≥ 4.5 kg; birthweight ≥ 90th centile; and each cardiometabolic outcome at the 9–11 year follow-up. In the fully adjusted model, birthweight ≥ 4.5 kg (Table 5) and birthweight ≥ 90th centile (Additional file 3: Table 3) were positively associated with weight z-score (B = 0.325, 95% CI = 0.018, 0.633, *p* = 0.038; B = 0.241, 95% CI = 0.015, 0.467, *p* = 0.037), height (cm) (B = 2.552, 95% CI = 0.589, 4.516, *p* = 0.011; B = 2.277, 95% CI = 0.841, 3.713, *p* = 0.002), height z-score (B = 0.391, 95% CI = 0.079, 0.703, *p* = 0.014; B = 0.373, 95% CI = 0.145, 0.600, *p* = 0.001), and lean mass (kg) (B = 1.353, 95% CI = 0.264, 2.442, *p* = 0.015; B = 1.005, 95% CI = 0.204, 1.805, *p* = 0.014), respectively. In the fully adjusted model, birthweight ≥ 90th centile was negatively associated with subscapular/triceps ratio (B = − 0.077, 95% CI = − 0.126, − 0.028, *p* = 0.002) (Additional file 3: Table 3). In the fully adjusted model, birthweight ≥ 4 kg was negatively associated with C3 concentration (g/L) (B = − 0.095, 95% CI = − 0.162, − 0.029, *p* = 0.005) and birthweight ≥ 4.5 kg was positively associated with cardiorespiratory endurance (20-m shuttle run test score) (B = 0.407, 95% CI = 0.006, 0.808, *p* = 0.047) (Table 6). There were no significant associations found between birthweight ≥ 90th centile and cardiometabolic outcomes (Additional file 4: Table 4). Sensitivity analyses yielded similar conclusions after further adjustment for preteen physical activity and sexual development in all models (Additional file 5: Table 5 and Additional file 6: Table 6).

**Table 5:** Multiple linear regression models between macrosomia and preteen anthropometry and body composition

	Model 1				Model 2				Model 3			
	B	95% CI	R^2^ adj	*p*	B	95% CI	R^2^ adj	*p*	B	95% CI	R^2^ adj	*p*
*Models for birthweight* ≥ *4 kg*												
Weight (kg)	0.099	(1.871, 2.069)	− 0.003	0.921	0.931	(− 0.791, 2.653)	0.292	0.288	0.732	(− 0.999, 2.463)	0.295	0.406
Weight z-score	0.181	(− 0.044, 0.407)	0.005	0.115	0.155	(− 0.069, 0.380)	0.090	0.175	0.128	(− 0.098, 0.353)	0.095	0.266
Height (cm)	0.086	(− 1.676, 1.847)	− 0.003	0.924	1.135	(− 0.297, 2.566)	0.388	0.120	1.033	(− 0.412, 2.478)	0.385	0.161
Height z-score	0.180	(− 0.039, 0.400)	0.005	0.107	0.176	(− 0.051, 0.404)	0.017	0.128	0.162	(− 0.067, 0.392)	0.011	0.166
BMI (kg/m^2^)	0.040	(− 0.614, 0.695)	− 0.003	0.904	0.178	(− 0.441, 0.797)	0.172	0.572	0.104	(− 0.517, 0.726)	0.176	0.742
BMI z-score	0.148	(− 0.100, 0.396)	0.001	0.241	0.111	(− 0.133, 0.355)	0.110	0.371	0.080	(− 0.165, 0.324)	0.117	0.520
MUAC (cm)	− 0.033	(− 0.725, 0.659)	− 0.003	0.925	0.223	(− 0.427, 0.873)	0.183	0.500	0.148	(− 0.505, 0.800)	0.187	0.657
WC (cm)	− 0.664	(− 2.646, 1.317)	− 0.002	0.510	− 0.303	(− 2.162, 1.556)	0.186	0.748	− 0.550	(− 2.413, 1.313)	0.193	0.562
Sum of skinfolds (mm)	− 1.754	(− 4.799, 1.291)	0.001	0.258	− 0.283	(− 3.176, 2.610)	0.168	0.847	− 0.807	(− 3.683, 2.069)	0.189	0.581
Subscapular/triceps ratio	− 0.040	(− 0.088, 0.008)	0.006	0.098	− 0.040	(− 0.089, 0.009)	0.051	0.107	− 0.043	(− 0.093, 0.006)	0.046	0.084
Lean mass (kg)	0.504	(− 0.450, 1.459)	0.000	0.299	0.699	(− 0.095, 1.492)	0.362	0.084	0.642	(− 0.158, 1.442)	0.360	0.115
Body fat (%)	− 0.915	(− 2.641, 0.811)	0.000	0.298	0.105	(− 1.509, 1.719)	0.193	0.899	− 0.265	(− 1.855, 1.324)	0.228	0.743
*Models for birthweight* ≥ *4.5 kg*												
Weight (kg)	1.628	(− 1.049, 4.304)	0.001	0.232	2.236	(− 0.125, 4.597)	0.298	0.063	2.163	(− 0.196, 4.522)	0.301	0.072
Weight z-score	0.397	(0.092, 0.703)	0.019	0.011	0.338	(0.030, 0.646)	0.098	0.032	0.325	(0.018, 0.633)	0.105	0.038
Height (cm)	1.228	(− 1.166, 3.623)	0.000	0.313	2.586	(0.630, 4.542)	0.397	0.010	2.552	(0.589, 4.516)	0.395	0.011
Height z-score	0.351	(0.053, 0.649)	0.015	0.021	0.395	(0.085, 0.706)	0.031	0.013	0.391	(0.079, 0.703)	0.026	0.014
BMI (kg/m^2^)	0.501	(− 0.388, 1.391)	0.001	0.268	0.462	(− 0.389, 1.313)	0.175	0.286	0.432	(− 0.418, 1.282)	0.178	0.318
BMI z-score	0.353	(0.016, 0.689)	0.011	0.040	0.230	(− 0.105, 0.565)	0.114	0.178	0.215	(− 0.119, 0.549)	0.121	0.207
MUAC (cm)	0.320	(− 0.622, 1.261)	− 0.002	0.505	0.393	(− 0.501, 1.287)	0.183	0.388	0.355	(− 0.538, 1.248)	0.188	0.435
WC (cm)	0.757	(− 1.942, 3.455)	− 0.002	0.581	0.738	(− 1.820, 3.297)	0.186	0.570	0.628	(− 1.923, 3.180)	0.193	0.628
Sum of skinfolds (mm)	0.297	(− 3.858, 4.453)	− 0.003	0.888	1.613	(− 2.365, 5.592)	0.170	0.425	1.402	(− 2.535, 5.338)	0.189	0.484
Subscapular/triceps ratio	− 0.040	(− 0.105, 0.025)	0.002	0.229	− 0.053	(− 0.120, 0.015)	0.050	0.124	− 0.053	(− 0.121, 0.014)	0.044	0.120
Lean mass (kg)	1.216	(− 0.078, 2.510)	0.008	0.065	1.354	(0.267, 2.441)	0.369	0.015	1.353	(0.264, 2.442)	0.368	0.015
Body fat (%)	− 0.296	(− 2.650, 2.059)	− 0.003	0.805	0.253	(− 1.969, 2.474)	0.193	0.823	0.132	(− 2.044, 2.308)	0.228	0.905

Models carried out between macrosomia and anthropometry and body composition outcomes at 9–11 years. *CI* Confidence interval, *BMI* Body mass index, *MUAC* Mid-upper arm circumference, *WC* Waist circumference. Model 1: crude results; Model 2: adjusted for age at follow-up, study group allocation, sex, HP index, maternal age at delivery, maternal ethnicity, maternal early pregnancy BMI, gestational weight gain, maternal smoking in pregnancy; Model 3: adjusted for breastfeeding exposure

**Table 6:** Multiple linear regression models between macrosomia and preteen cardiometabolic outcomes

	Model 1				Model 2				Model 3			
	B	95% CI	R^2^ adj	*p*	B	95% CI	R^2^ adj	*p*	B	95% CI	R^2^ adj	*p*
*Models for birthweight* ≥ *4 kg*												
SBP percentile	0.939	(− 4.336, 6.214)	− 0.003	0.726	0.746	(− 4.793, 6.284)	− 0.020	0.791	0.238	(− 5.303, 5.779)	− 0.007	0.933
DBP percentile	− 1.658	(− 7.059, 3.742)	− 0.002	0.546	− 0.834	(− 6.487, 4.819)	− 0.013	0.772	− 1.231	(− 6.929, 4.466)	− 0.015	0.671
Resting heart rate (bpm)	0.725	(− 2.184, 3.634)	− 0.003	0.624	1.520	(− 1.511, 4.550)	− 0.004	0.324	1.681	(− 1.363, 4.725)	0.001	0.278
20-M SRT score	0.171	(− 0.137, 0.478)	0.001	0.275	0.170	(− 0.136, 0.477)	0.085	0.274	0.203	(− 0.090, 0.497)	0.171	0.174
HOMA-IR	− 0.199	(− 0.984, 0.585)	− 0.004	0.617	− 0.313	(− 1.117, 0.491)	0.028	0.443	− 0.356	(− 1.150, 0.437)	0.067	0.376
TC (mmol/L)	− 0.131	(− 0.339, 0.077)	0.003	0.214	− 0.115	(− 0.333, 0.103)	− 0.010	0.297	− 0.128	(− 0.346, 0.091)	− 0.002	0.250
Triglycerides (mmol/L)	− 0.146	(− 0.332, 0.040)	0.008	0.123	− 0.145	(− 0.339, 0.050)	0.001	0.143	− 0.150	(− 0.343, 0.043)	0.031	0.126
LDL-C (mmol/L)	− 0.027	(− 0.195, 0.140)	− 0.005	0.748	− 0.024	(− 0.199, 0.152)	− 0.017	0.791	− 0.034	(− 0.210, 0.143)	− 0.011	0.705
HDL-C (mmol/L)	− 0.042	(− 0.155, 0.072)	− 0.003	0.470	− 0.029	(− 0.147, 0.089)	− 0.004	0.631	− 0.029	(− 0.147, 0.089)	0.007	0.630
C-reactive protein (mg/L)	− 0.097	(− 0.468, 0.274)	− 0.004	0.607	− 0.062	(− 0.455, 0.331)	− 0.037	0.757	− 0.071	(− 0.453, 0.312)	0.030	0.716
C3 complement (g/L)	− 0.070	(− 0.145, 0.005)	0.014	0.065	− 0.080	(− 0.156, − 0.005)	0.072	0.037	− 0.095	(− 0.162, − 0.029)	0.291	0.005
ICAM-1 (pg/mL)^a^	− 0.044	(− 0.091, 0.002)	0.019	0.062	− 0.042	(− 0.090, 0.007)	0.021	0.093	− 0.042	(− 0.091, 0.008)	0.008	0.097
TNF-α (pg/mL)	− 0.525	(− 1.453, 0.402)	0.002	0.265	− 0.624	(− 1.580, 0.333)	0.022	0.199	− 0.716	(− 1.670, 0.238)	0.041	0.140
GDF-15 (pg/mL)^a^	− 0.041	(− 0.091, 0.009)	0.012	0.105	− 0.032	(− 0.085, 0.021)	− 0.039	0.237	− 0.035	(− 0.089, 0.019)	− 0.054	0.196
sCD163 (pg/mL)^a^	− 0.037	(− 0.091, − 0.018)	0.006	0.183	− 0.041	(− 0.096, 0.013)	0.068	0.137	− 0.041	(− 0.096, 0.014)	0.080	0.143
Leptin (pg/mL)^a^	− 0.030	(− 0.186, 0.125)	− 0.006	0.701	0.005	(− 0.144, 0.155)	0.140	0.944	− 0.017	(− 0.123, 0.089)	0.573	0.751
IL-6 (pg/mL)	− 0.013	(− 0.309, 0.283)	− 0.008	0.931	0.058	(− 0.238, 0.354)	0.071	0.699	0.049	(− 0.247, 0.346)	0.079	0.743
IL-17A (pg/mL)	− 0.239	(− 0.609, 0.132)	0.005	0.205	− 0.293	(− 0.665, 0.079)	0.072	0.122	− 0.270	(− 0.648, 0.107)	0.061	0.158
*Models for birthweight* ≥ *4.5 kg*												
SBP percentile	2.585	(− 4.593, 9.763)	− 0.002	0.479	2.443	(− 5.177, 10.063)	− 0.019	0.529	1.678	(− 5.919, 9.274)	− 0.007	0.664
DBP percentile	− 6.124	(13.449, 1.200)	0.006	0.101	− 5.878	(13.631, 1.875)	− 0.006	0.137	− 6.463	(− 14.242, 1.315)	− 0.006	0.103
Resting heart rate (bpm)	− 0.731	(− 4.693, 3.231)	− 0.003	0.717	0.787	(− 3.391, 4.965)	− 0.007	0.711	0.578	(− 3.604, 4.761)	− 0.003	0.786
20-M SRT score	0.213	(− 0.206, 0.631)	0.000	0.318	0.326	(− 0.095, 0.747)	0.089	0.128	0.407	(0.006, 0.808)	0.177	0.047
HOMA-IR	0.560	(− 0.506, 1.626)	0.000	0.301	0.440	(− 0.667, 1.546)	0.028	0.434	0.338	(− 0.751, 1.428)	0.064	0.541
TC (mmol/L)	− 0.146	(− 0.430, 0.137)	0.000	0.311	− 0.058	(− 0.359, 0.243)	− 0.016	0.703	− 0.069	(− 0.369, 0.232)	− 0.009	0.652
Triglycerides (mmol/L)	0.123	(− 0.131, 0.377)	0.000	0.340	0.144	(− 0.124, 0.412)	− 0.005	0.290	0.125	(− 0.140, 0.390)	0.022	0.354
LDL-C (mmol/L)	− 0.183	(− 0.410, 0.044)	0.009	0.114	− 0.143	(− 0.384, 0.098)	− 0.009	0.244	− 0.149	(− 0.390, 0.092)	− 0.002	0.225
HDL-C (mmol/L)	− 0.035	(− 0.190, 0.120)	− 0.005	0.655	0.004	(− 0.59, 0.167)	− 0.006	0.964	0.008	(− 0.154, 0.170)	0.006	0.923
C-reactive protein (mg/L)	0.001	(− 0.505, 0.507)	− 0.006	0.996	− 0.005	(− 0.546, 0.536)	− 0.038	0.986	− 0.073	(− 0.598, 0.452)	0.029	0.785
C3 complement (g/L)	− 0.030	(− 0.133, 0.072)	− 0.004	0.560	− 0.051	(− 0.156, 0.054)	0.053	0.340	− 0.074	(− 0.166, 0.019)	0.266	0.118
ICAM-1 (pg/mL)^a^	− 0.038	(− 0.102, 0.026)	0.003	0.240	− 0.046	(− 0.114, 0.021)	0.013	0.174	− 0.049	(− 0.116, 0.019)	0.001	0.159
TNF-α (pg/mL)	− 0.116	(− 1.386, 1.153)	− 0.007	0.856	− 0.098	(− 1.424, 1.228)	0.009	0.884	− 0.207	(− 1.527, 1.113)	0.024	0.757
GDF-15 (pg/mL)^a^	− 0.020	(− 0.088, 0.048)	− 0.005	0.565	− 0.004	(− 0.077, 0.070)	− 0.051	0.918	− 0.005	(− 0.080, 0.069)	− 0.068	0.891
sCD163 (pg/mL)^a^	− 0.012	(− 0.087, 0.062)	− 0.007	0.744	− 0.033	(− 0.109, 0.043)	0.057	0.388	− 0.035	(− 0.111, 0.040)	0.070	0.358
Leptin (pg/mL)^a^	− 0.019	(− 0.230, 0.193)	− 0.007	0.862	− 0.036	(− 0.242, 0.170)	0.141	0.731	− 0.089	(− 0.234, 0.056)	0.578	0.226
IL-6 (pg/mL)	− 0.134	(− 0.536, 0.268)	− 0.004	0.510	− 0.105	(− 0.512, 0.302)	0.072	0.610	− 0.136	(− 0.542, 0.270)	0.082	0.508
IL-17A (pg/mL)	− 0.152	(− 0.658, 0.355)	− 0.005	0.555	− 0.191	(− 0.708, 0.325)	0.057	0.465	− 0.188	(− 0.709, 0.332)	0.049	0.475

Models carried out as macrosomia and cardiometabolic outcomes at 9–11 years. ^a^log10 transformed data was used. *CI* Confidence interval, *SBP* Systolic blood pressure, *DBP* Diastolic blood pressure, *20-M SRT* 20-m shuttle run test, *HOMA-IR* Homeostatic Model Assessment for Insulin Resistance, *TC* Total cholesterol, *LDL-C* Low density lipoprotein cholesterol, *HDL-C* High density lipoprotein cholesterol, *ICAM-1* Intracellular adhesion molecule 1, *TNF-α* Tumour necrosis factor alpha, *GDF-15* Growth differentiation factor 15, *sCD163* Soluble cluster of differentiation factor 163, *IL* Interleukin. Model 1: crude results; Model 2: adjusted for age at follow-up, study group allocation, sex, HP index, maternal age at delivery, maternal ethnicity, maternal early pregnancy BMI, gestational weight gain, maternal smoking in pregnancy; Model 3: adjusted for breastfeeding exposure, preteen BMI

## Discussion

This exploratory study found no convincing evidence to suggest that macrosomia is linked to the programming of adverse cardiometabolic health at 9–11 years of age. Preteens born with macrosomia had higher weight z-scores, higher BMI z-scores, were taller, and leaner, and had lower diastolic blood pressure percentile and lower inflammation, compared to those born without macrosomia. The results of the adjusted regression analyses revealed that high birthweight was associated with a taller and leaner body composition, along with lower inflammation and higher fitness at 9–11 years of age.

The pathophysiology linking high birthweight and cardiometabolic risk in later life remains unknown, highlighting the importance of this research. Based on previous research, we expected that early precursors of obesity and cardiometabolic disease would be evident in youth born with macrosomia at 9–11 years of age [20, 24, 25]. Despite this, the prevalence of overweight and obesity in this preteen cohort did not differ between groups and was similar to an unselected cohort of similar age from the Growing Up in Ireland study [48]. This might, at least in part, account for the lack of long-term impact on cardiometabolic risk factors observed in preteens with a birthweight ≥ 4 kg relative to their counterparts born < 4 kg. This is important, given 1 in 5 neonates weigh greater than 4 kg at birth in some regions and more than 60% of neonates with macrosomia are born to women without identifiable risk factors [49, 50].

We found differences in body composition at 9–11 years of age between those born with and without macrosomia. Those born ≥ 4.5 kg had higher weight and height z-scores, were taller, leaner, and had higher BMI z-scores at 9–11 years compared to those born < 4.5 kg. Similar body composition profiles were observed amongst those born with a birthweight above the 90th centile. Thus, it is plausible those at the upper end of the macrosomic range may have a higher risk of obesity and metabolic syndrome in later life. In adjusted analyses, high birthweight was positively associated with weight and height z-scores, along with lean mass at 9–11 years of age. Previous studies have shown that larger neonates maintain a tall and lean profile into adolescence and adulthood, also displaying higher bone density and muscle mass in old age [16, 51]. This may also explain our finding of a positive association between high birthweight and fitness in adjusted analyses [52]. It is also important to acknowledge the exploratory nature of this study, which may limit the interpretation of our results.

Postnatal growth patterns can independently influence later body composition and those outside the healthy birthweight range tend to return to their genetically determined growth trajectory within the first two years of life [53]. Maternal influence in utero may be compensated by patterns of “catch-up” and “catch-down” growth for low birthweight and high birthweight infants respectively [54]. While the proportion and timing remains unclear, the “catch-down” growth phase has shown protective effects on body composition in 8-year-old children exposed to excess intrauterine fetal growth [55]. Despite this, an estimated 20% of high birthweight infants who do not enter “catch-down” growth represent a high-risk subgroup and maintain higher subcutaneous fat and BMI in later childhood [53]. Lurbe et al. [2] also found that weight gain in early life may also override the effects of high birthweight on obesity and metabolic risk at 10-years of age. Earlier adiposity rebound in children born with high birthweight is another potential risk factor for obesity [9]. Therefore, postnatal growth may be a critical factor in determining the long-term risks associated with macrosomia in our high birthweight cohort beyond infancy [54]. Additionally, the time point of our investigation may influence the differences observed between the groups, because the preteen period can be a transitional phase for body composition profiles in both sexes [56].

The assessment of cardiometabolic health in children is challenging and our novel study included a broad range of cardiometabolic indicators to provide greater insight into metabolic and inflammatory processes. In adjusted analyses, there was only a weak association between macrosomia and lower inflammation (C3 complement protein). Our findings are consistent with results from a small study (n = 90) which found high birthweight was not related to blood pressure or lipid profiles [25]. Sparano et al. [10] also reported no significant impact of macrosomia on blood pressure, lipids, glucose, insulin, HOMA-IR, and HbA1c in 7-year-old European children. Significant associations have been found in adolescent and adult cohorts [18, 20, 21]. It is possible that the high variability of metabolic parameters in children < 10 years of age may limit the ability to detect consequences of macrosomia on biochemical parameters. Alternative assessment by echocardiography may also provide clearer results. Recently, Yapicioglu et al. [24] found 8–9-year-olds born with macrosomia have higher carotid intima-media thickness levels than those born without macrosomia.

Our findings of no relationship between macrosomia and increased cardiometabolic risk in preteens may be explained by the role of the postnatal lifestyle and biological factors that can modify or add to the risks established during intrauterine development [40, 42, 45, 46, 57], many of these we were able to control for within our analyses. It is challenging to establish causality linking fetal life with preteen cardiometabolic health, and various factors after birth may play a role. Correlated aspects of the postnatal environment are often overlooked in studies that focus on birthweight. This study attempted to further tease apart the possible effects of postnatal lifestyle and biological factors in sensitivity analyses, however, preteen physical activity and sexual maturity had minimal effects. The weak influence of different intrauterine factors including maternal glycaemia and metabolic parameters on child adiposity was also shown in the ROLO cohort up to 5-years of age [58, 59]. In contrast, other analyses in the ROLO cohort have reported associations between possible programming of early childhood health and several maternal dietary exposures in utero [60–63]. Therefore, additional research is needed to elucidate the perinatal etiology of cardiometabolic diseases to prevent negative consequences manifesting in childhood. Further research may also explore associations between perinatal exposures and composite cardiometabolic risk scores in youth. Rather than examining risk factors individually, a combined approach may better reflect metabolic patterns and have greater potential to translate to a larger public health impact [64].

This study is strengthened by the longitudinal design and unique Irish cohort who were born to mothers that previously delivered an infant with macrosomia and over half were born with recurrent macrosomia. Additional strengths include a large sample size of preteens with 53.3% follow-up (n = 405) of the original 759 ROLO mother-child dyads, which has been steadily maintained since the 5-year follow-up [59]. Detailed measures of adiposity, cardiovascular health, and cardiorespiratory endurance enables a thorough assessment of preteen cardiometabolic health. In addition, complete data is available for a large proportion of the cohort. The inclusion of traditional and non-traditional serum biomarkers for a subgroup of preteens provides valuable insight into endothelial, metabolic, and inflammatory processes. Majority of research in children and adults rely on BMI, and our analysis contributes to a gap in our understanding of the programming of fat and fat-free mass by including DXA measures [11, 13, 49, 65].

This analysis has several limitations. The serum samples were obtained in a non-fasting state which may not provide an accurate reflection of metabolism. In the literature, the criteria defining high birthweight criteria can vary, which may also complicate direct comparison between studies. The 9–11 year follow-up was conducted over a 6-year period resulting in differences in age and pubertal status, however, this was addressed by including these factors in adjusted analyses. Differences in blood pressure results may have been more apparent with the use of 24 h blood pressure monitoring. Primary sexual development in girls was accounted for by self-report of breast development, however, only self-report of adrenarche was sought in boys rather than testicular volume. This exploratory study is also unable to account for factors that were not included as potential confounders which may impact the interpretation of findings. Given the large number of statistical tests run for this analysis, significant results may be chance findings and should be interpreted with caution. One avenue of future research that we did not explore in detail should focus on potential sex-specific effects of programming on preteen cardiometabolic indicators, given the strong genetic influence on cardiometabolic risk between males and females [66].

## Conclusion

Our novel longitudinal study found no convincing evidence to suggest that macrosomia is associated with adverse preteen cardiometabolic outcomes, minimising the potential longitudinal impact of this risk factor. Additional longitudinal research may further explore the influence of postnatal factors such as growth, early feeding, and lifestyle in relation to size at birth to better understand the early life origins of obesity and metabolic disease.

### Supplementary Information

**Additional file 1**. Differences in baseline characteristics between the original ROLO pregnancy cohort and the follow-up cohort at 9–11 years

**Additional file 2**. Comparison of cardiometabolic health in 9–11-year-old preteens born with a birthweight ≥90th and <90th centile

**Additional file 3**. Multiple linear regression models between birthweight centile and preteen anthropometry and body composition

**Additional file 4**. Multiple linear regression models between birthweight centile and preteen cardiometabolic outcomes

**Additional file 5**. Sensitivity analyses between macrosomia and preteen anthropometry and body composition

**Additional file 6**. Sensitivity analyses between macrosomia and preteen cardiometabolic outcomes

## Data Availability

The datasets used and/or analysed during the current study are available from the corresponding author on reasonable request.

## Abbreviations

ROLO: Randomised cOntrol trial of a LOw glycaemic index diet in pregnancy to prevent macrosomia
BMI: Body mass index
DXA: Dual-energy x-ray absorptiometry
HDL-C: High density lipoprotein cholesterol
C3: Complement protein
HOMA-IR: Homeostatic Model Assessment for Insulin Resistance
LDL-C: Low density lipoprotein cholesterol
ICAM-1: Intracellular adhesion molecule 1
TNF-α: Tumour necrosis factor alpha
GDF-15: Growth differentiation factor 15
sCD163: Soluble cluster of differentiation factor 163
IL: Interleukin
PAQ-C: Physical Activity Questionnaire for Older Children
SD: Standard deviation
IQR: Interquartile range
