# Supplementary material for: The impact of macrosomia on cardiometabolic health in preteens: findings from the ROLO longitudinal birth cohort study
Source: Nutr Metab (Lond). 2023 Sep 4;20:37. doi: 10.1186/s12986-023-00759-8 (PMC10476328; doi:10.1186/s12986-023-00759-8)
Supplement: Supplementary file 1 — Additional file 1. Differences in baseline characteristics between the original ROLO pregnancy cohort and the follow-up cohort at 9–11 years [file 12986_2023_759_MOESM1_ESM.docx]

| Supplementary Table 1. Differences in baseline characteristics between the original ROLO pregnancy cohort and the follow-up cohort at 9-11 years. | | | | | | | |
| --- | --- | --- | --- | --- | --- | --- | --- |
|  | **Follow-up cohort at 9-11 years**  **(n = 405)** | | | **Original ROLO pregnancy cohort**  **(n = 759)** | | |  |
|  | N | Median/n | (IQR)/% | N | Median/n | (IQR)/% | *p* |
| Maternal characteristics | | | | | | | |
| Age at delivery (years) | 404 | 33.17 | (30.5, 35.47) | 755 | 32.61 | (29.76, 35.41) | 0.091 |
| Early pregnancy BMI (kg/m^2^) | 401 | 25.46 | (23.3, 27.96) | 755 | 25.67 | (23.37, 28.8) | 0.222 |
| HP index | 405 | 7.5 | (1.1, 12.5) | 755 | 6.9 | (-0.5, 12.5) | 0.150 |
| Gestational weight gain |  |  |  |  |  |  |  |
| Inadequate, n (%) | 333 | 52 | 15.6 | 628 | 94 | 15.0 | 0.620 |
| Adequate, n (%) | 333 | 127 | 38.1 | 628 | 223 | 35.5 |  |
| Excessive, n (%) | 333 | 154 | 46.2 | 628 | 311 | 49.5 |  |
| Ethnicity (White Irish), n (%) | 405 | 372 | 91.9 | 759 | 691 | 91.0 | 0.640 |
| Smoking in pregnancy, n (%) | 405 | 13 | 3.2 | 759 | 30 | 4.0 | 0.522 |
| RCT group (intervention), n (%) | 405 | 203 | 50.1 | 759 | 369 | 48.6 | 0.624 |
| Infant characteristics | | | | | | | |
| Child sex (male), n (%) | 405 | 203 | 50.1 | 756 | 376 | 49.7 | 0.900 |
| Birthweight (kg) | 405 | 4.01 | (3.73, 4.34) | 759 | 4.0 | (3.73, 4.33) | 0.875 |
| Birthweight centile | 404 | 86.32 | (72.23, 95.68) | 753 | 86.19 | (71.14, 95.71) | 0.943 |
| Gestational age at delivery (days) | 404 | 283.0 | (277.0, 288.0) | 754 | 282.5 | (277.0, 288.0) | 0.882 |
| Birthweight ≥4 kg, n (%) | 405 | 208 | 51.4 | 759 | 388 | 51.4 | 0.992 |
| Birthweight ≥4.5 kg, n (%) | 405 | 65 | 16.0 | 759 | 119 | 15.8 | 0.898 |
| Birthweight ≥90^th^ centile, n (%) | 404 | 159 | 39.4 | 753 | 296 | 39.3 | 0.988 |
| Results presented as median (IQR 25^th^ – 75^th^ percentile) for non-normally distributed variables. N = total population with available data; n = frequency. Abbreviations: ROLO Randomised cOntrol trial of LOw glycaemic index diet in pregnancy versus no dietary intervention to prevent recurrence of macrosomia; IQR Interquartile range; BMI Body mass index; HP index Hasse and Pratschke Deprivation index; RCT Randomised control trial. *P* values determined using Mann-Whitney U tests for non-normally distributed variables; Chi square tests for categorical variables. | | | | | | | |
