# Supplementary material for: The impact of macrosomia on cardiometabolic health in preteens: findings from the ROLO longitudinal birth cohort study
Source: Nutr Metab (Lond). 2023 Sep 4;20:37. doi: 10.1186/s12986-023-00759-8 (PMC10476328; doi:10.1186/s12986-023-00759-8)
Supplement: Supplementary file 2 — Additional file 2. Comparison of cardiometabolic health in 9–11-year-old preteens born with a birthweight ≥90th and <90th centile [file 12986_2023_759_MOESM2_ESM.docx]

| Supplementary Table 2. Comparison of cardiometabolic health in 9-11-year-old preteens born with a birthweight ≥90^th^ and <90^th^ centile. | | | | | | | |
| --- | --- | --- | --- | --- | --- | --- | --- |
|  | **Birthweight ≥90^th^ centile** | | | **Birthweight <90^th^ centile** | | |  |
|  | N | Mean/Median/n | SD/(IQR)/% | N | Mean/Median/n | SD/(IQR)/% | *p* |
| Anthropometry and body composition | | | | | | | |
| Weight (kg) | 159 | 34.4 | (31.6, 40.2) | 245 | 33.6 | (29.7, 39.8) | 0.059 |
| Weight z-score | 159 | 0.7 | 0.92 | 245 | 0.44 | 1.01 | 0.010 |
| Height (cm) | 159 | 141.4 | (136.8, 145.8) | 245 | 139.1 | (134.4, 146.6) | 0.028 |
| Height z-score | 159 | 0.73 | 0.91 | 245 | 0.38 | 0.96 | <0.001 |
| BMI (kg/m^2^) | 159 | 17.44 | (16.12, 19.48) | 245 | 17.14 | (15.63, 19.65) | 0.192 |
| BMI z-score | 159 | 0.49 | 1.05 | 245 | 0.36 | 1.09 | 0.209 |
| Overweight, n (%) | 159 | 40 | 25.2 | 245 | 46 | 18.8 | 0.481 |
| Obesity, n (%) | 159 | 12 | 7.5 | 245 | 21 | 8.6 |  |
| MUAC (cm) | 158 | 21.2 | (19.4, 23.42) | 242 | 20.8 | (19.1, 23.2) | 0.221 |
| WC (cm) | 159 | 62.5 | (58.9, 67.2) | 245 | 62.0 | (58.4, 68.1) | 0.902 |
| Sum of skinfolds (mm) | 143 | 28.0 | (21.93, 37.96) | 222 | 28.53 | (20.91, 36.5) | 0.634^a^ |
| Subscapular/triceps ratio | 146 | 0.57 | (0.47, 0.67) | 228 | 0.6 | (0.51, 0.75) | 0.004 |
| Lean mass (kg) | 137 | 24.56 | (22.95, 27.23) | 210 | 23.7 | (21.75, 26.89) | 0.015 |
| Body fat (%) | 137 | 26.4 | (20.85, 32.05) | 210 | 26.3 | (20.97, 31.9) | 0.409^a^ |
| Cardiovascular health and cardiorespiratory endurance | | | | | | | |
| SBP percentile | 149 | 85.0 | (72.0, 95.0) | 230 | 89.0 | (66.75, 96.0) | 0.770 |
| DBP percentile | 149 | 73.0 | (49.5, 85.0) | 230 | 73.5 | (54.0, 88.0) | 0.552 |
| Resting heart rate (bpm) | 147 | 78.95 | 12.76 | 229 | 79.09 | 12.63 | 0.917 |
| 20-M SRT score | 151 | 3.5 | (3.1, 4.9) | 226 | 3.6 | (3.2, 4.5) | 0.913 |
| Cardiometabolic biomarkers | | | | | | | |
| HOMA-IR | 88 | 2.0 | (1.31, 4.45) | 122 | 3.06 | (1.57, 5.34) | 0.037^a^ |
| TC (mmol/L) | 88 | 4.13 | 0.73 | 124 | 4.17 | 0.67 | 0.715 |
| Triglycerides (mmol/L) | 89 | 0.87 | (0.67, 1.2) | 124 | 0.89 | (0.69, 1.46) | 0.562 |
| LDL-C (mmol/L) | 88 | 2.41 | 0.58 | 124 | 2.41 | 0.54 | 0.988 |
| HDL-C (mmol/L) | 89 | 1.24 | 0.37 | 124 | 1.24 | 0.38 | 0.977 |
| C-reactive protein (mg/L) | 84 | 0.25 | (0.13, 0.43) | 118 | 0.22 | (0.11, 0.5) | 0.676^a^ |
| C3 complement (g/L) | 89 | 1.38 | (1.21, 1.55) | 124 | 1.39 | (1.24, 1.6) | 0.247^a^ |
| ICAM-1 (ng/mL) | 65 | 345.97 | (291.39, 394.99) | 98 | 375.15 | (303.7, 436.87) | 0.053 |
| TNF-α (pg/mL) | 65 | 8.72 | 2.39 | 98 | 8.59 | 2.92 | 0.772 |
| GDF-15 (ng/mL) | 65 | 0.29 | (0.25, 0.34) | 98 | 0.29 | (0.24, 0.35) | 0.964 |
| sCD163 (ng/mL) | 65 | 428.66 | (346.08, 552.59) | 97 | 430.1 | (332.55, 558.77) | 0.938^a^ |
| Leptin (ng/mL) | 65 | 5.16 | (1.59, 9.09) | 98 | 3.97 | (1.73, 8.52) | 0.614 |
| IL-6 (pg/mL) | 64 | 0.7 | (0.47, 1.04) | 98 | 0.76 | (0.49, 1.11) | 0.698^a^ |
| IL-17A (pg/mL) | 65 | 1.06 | (0.73, 2.01) | 98 | 1.33 | (0.97, 2.18) | 0.038 |
| Results presented as mean (SD) for normally distributed variables, median (IQR 25^th^ – 75^th^ percentile) for non-normally distributed variables, and n (%) for categorical variables. N = total population with available data; n = frequency. ^a^log10 transformed data was used. Abbreviations: SD Standard deviation; IQR Interquartile range; BMI Body mass index; MUAC Mid-upper arm circumference; WC Waist circumference; SBP Systolic blood pressure; DBP Diastolic blood pressure; 20-M SRT 20-metre shuttle run test; HOMA-IR Homeostatic Model Assessment for Insulin Resistance; TC Total cholesterol; LDL-C Low density lipoprotein cholesterol; HDL-C High density lipoprotein cholesterol; ICAM-1 Intracellular adhesion molecule 1; TNF-α Tumour necrosis factor alpha; GDF-15 Growth differentiation factor 15; sCD163 Soluble cluster of differentiation factor 163; IL Interleukin. *P* values determined using independent *t*-tests for normally distributed variables; Mann-Whitney U tests for non-normally distributed variables; Chi square tests for categorical variables. | | | | | | | |
