# Supplementary material for: The impact of macrosomia on cardiometabolic health in preteens: findings from the ROLO longitudinal birth cohort study
Source: Nutr Metab (Lond). 2023 Sep 4;20:37. doi: 10.1186/s12986-023-00759-8 (PMC10476328; doi:10.1186/s12986-023-00759-8)
Supplement: Supplementary file 3 — Additional file 3. Multiple linear regression models between birthweight centile and preteen anthropometry and body composition [file 12986_2023_759_MOESM3_ESM.docx]

| Supplementary Table 3. Multiple linear regression models between birthweight centile and preteen anthropometry and body composition. | | | | | | | | | | | | | | | |
| --- | --- | --- | --- | --- | --- | --- | --- | --- | --- | --- | --- | --- | --- | --- | --- |
|  | **Model 1** | | | | **Model 2** | | | | | **Model 3** | | | | | |
|  | B | 95% CI | R^2^ Adj | *p* | B | 95% CI | R^2^ Adj | *p* | | B | 95% CI | R^2^ Adj | *p* | |  |
| Models for birthweight ≥90^th^ centile | | | | | | | | | | | | | | | |
| Weight (kg) | 1.107 | (-0.905, 3.118) | 0.001 | 0.280 | 1.568 | (-0.160, 3.296) | 0.298 | | 0.075 | 1.393 | (-0.345, 3.130) | 0.299 | | 0.116 | |
| Weight z-score | 0.258 | (0.028, 0.488) | 0.013 | 0.028 | 0.264 | (0.039, 0.489) | 0.101 | | 0.022 | 0.241 | (0.015, 0.467) | 0.105 | | 0.037 | |
| Height (cm) | 1.468 | (-0.326, 3.262) | 0.005 | 0.108 | 2.357 | (-0.935, 3.778) | 0.405 | | 0.001 | 2.277 | (0.841, 3.713) | 0.401 | | 0.002 | |
| Height z-score | 0.347 | (0.125, 0.569) | 0.028 | 0.002 | 0.382 | (0.157, 0.608) | 0.047 | | <0.001 | 0.373 | (0.145, 0.600) | 0.040 | | 0.001 | |
| BMI (kg/m^2^) | 0.224 | (-0.446, 0.893) | -0.002 | 0.511 | 0.226 | (-0.397, 0.849) | 0.173 | | 0.475 | 0.159 | (-0.467, 0.785) | 0.176 | | 0.617 | |
| BMI z-score | 0.139 | (-0.115, 0.393) | 0.001 | 0.284 | 0.123 | (-0.123, 0.368) | 0.111 | | 0.326 | 0.096 | (-0.150, 0.342) | 0.118 | | 0.445 | |
| MUAC (cm) | 0.375 | (-0.332, 1.081) | 0.000 | 0.298 | 0.420 | (-0.234, 1.073) | 0.186 | | 0.207 | 0.356 | (-0.300, 1.013) | 0.189 | | 0.286 | |
| WC (cm) | -0.386 | (-2.414, 1.642) | -0.003 | 0.708 | 0.035 | (-1.838, 1.908) | 0.185 | | 0.971 | -0.194 | (-2.071, 1.684) | 0.192 | | 0.839 | |
| Sum of skinfolds (mm) | 0.549 | (-2.753, 3.671) | -0.003 | 0.729 | 0.156 | (-2.758, 3.070) | 0.168 | | 0.916 | -0.342 | (-3.240, 2.555) | 0.188 | | 0.816 | |
| Subscapular/triceps ratio | -0.061 | (-0.109, -0.012) | 0.017 | 0.015 | -0.073 | (-0.121, -0.024) | 0.071 | | 0.003 | -0.077 | (-0.126, -0.028) | 0.067 | | 0.002 | |
| Lean mass (kg) | 0.783 | (-0.191, 1.757) | 0.005 | 0.115 | 1.058 | (0.264, 1.851) | 0.371 | | 0.009 | 1.005 | (0.204, 1.805) | 0.368 | | 0.014 | |
| Body fat (%) | 0.570 | (-1.198, 2.337) | -0.002 | 0.527 | 0.404 | (-1.221, 2.029) | 0.194 | | 0.625 | 0.052 | (-1.549, 1.652) | 0.228 | | 0.949 | |
| Models carried out as birthweight centile and anthropometry and body composition outcomes at 9-11 years. Abbreviations: CI Confidence interval; BMI Body mass index; MUAC Mid-upper arm circumference; WC Waist circumference. Model 1: crude results; Model 2: adjusted for age at follow-up, study group allocation, sex, HP index, maternal age at delivery, maternal ethnicity, maternal early pregnancy BMI, gestational weight gain, maternal smoking in pregnancy; Model 3: adjusted for breastfeeding exposure. | | | | | | | | | | | | | | | |
