# Supplementary material for: The impact of macrosomia on cardiometabolic health in preteens: findings from the ROLO longitudinal birth cohort study
Source: Nutr Metab (Lond). 2023 Sep 4;20:37. doi: 10.1186/s12986-023-00759-8 (PMC10476328; doi:10.1186/s12986-023-00759-8)
Supplement: Supplementary file 4 — Additional file 4. Multiple linear regression models between birthweight centile and preteen cardiometabolic outcomes [file 12986_2023_759_MOESM4_ESM.docx]

| Supplementary Table 4. Multiple linear regression models between birthweight centile and preteen cardiometabolic outcomes. | | | | | | | | | | | | |
| --- | --- | --- | --- | --- | --- | --- | --- | --- | --- | --- | --- | --- |
|  | **Model 1** | | | | **Model 2** | | | | **Model 3** | | | |
|  | B | 95% CI | R^2^ Adj | *p* | B | 95% CI | R^2^ Adj | *p* | B | 95% CI | R^2^ Adj | *p* |
| Models for birthweight ≥90^th^ centile | | | | | | | | | | | | |
| SBP percentile | 0.952 | (-4.445, 6.349) | -0.003 | 0.729 | 0.274 | (-5.304, 5.853) | -0.021 | 0.923 | -0.157 | (-5.739, 5.425) | -0.007 | 0.956 |
| DBP percentile | -0.956 | (-6.484, 4.571) | -0.003 | 0.734 | -1.402 | (-7.094, 4.290) | -0.013 | 0.628 | -1.798 | (-7.535, 3.938) | -0.014 | 0.538 |
| Resting heart rate (bpm) | -0.139 | (-3.117, 2.838) | -0.003 | 0.927 | 0.221 | (-2.836, 3.279) | -0.007 | 0.887 | 0.347 | (-2.725, 3.420) | -0.003 | 0.824 |
| 20-M SRT score | -0.017 | (-0.332, 0.299) | -0.003 | 0.918 | 0.084 | (-0.225, 0.393) | 0.082 | 0.593 | 0.124 | (-0.173, 0.420) | 0.167 | 0.412 |
| HOMA-IR | -0.627 | (-1.425, 0.171) | 0.008 | 0.123 | -0.536 | (-1.342, 0.271) | 0.035 | 0.192 | -0.600 | (-1.396, 0.195) | 0.075 | 0.138 |
| TC (mmol/L) | -0.036 | (-0.249, 0.178) | -0.005 | 0.741 | -0.025 | (-0.246, 0.195) | -0.017 | 0.820 | -0.042 | (-0.263, 0.179) | -0.009 | 0.707 |
| Triglycerides (mmol/L) | -0.090 | (-0.281, 0.101) | -0.001 | 0.353 | -0.112 | (-0.308, 0.084) | -0.004 | 0.260 | -0.122 | (-0.317, 0.072) | 0.026 | 0.216 |
| LDL-C (mmol/L) | 0.001 | (-0.171, 0.173) | -0.006 | 0.989 | 0.002 | (-0.175, 0.179) | -0.017 | 0.980 | -0.013 | (-0.191, 0.165) | -0.012 | 0.886 |
| HDL-C (mmol/L) | -0.002 | (-0.118, 0.115) | -0.006 | 0.979 | 0.018 | (-0.674, 1.798) | -0.005 | 0.371 | 0.020 | (-0.099, 0.140) | 0.007 | 0.735 |
| C-reactive protein (mg/L) | -0.023 | (-0.403, 0.357) | -0.006 | 0.906 | -0.079 | (-0.475, 0.316) | -0.037 | 0.693 | -0.086 | (-0.471, 0.300) | 0.030 | 0.662 |
| C3 complement (g/L) | -0.043 | (-0.120, 0.034) | 0.001 | 0.276 | -0.051 | (-0.127, 0.026) | 0.057 | 0.195 | -0.067 | (-0.135, 0.000) | 0.272 | 0.051 |
| ICAM-1 (pg/mL)^a^ | -0.029 | (-0.077, -0.019) | 0.003 | 0.241 | -0.033 | (-0.083, 0.016) | 0.012 | 0.181 | -0.034 | (-0.084, 0.016) | 0.000 | 0.181 |
| TNF-α (pg/mL) | 0.127 | (-0.827, 1.080) | -0.007 | 0.793 | 0.147 | (-0.823, 1.117) | 0.009 | 0.765 | 0.074 | (-0.896, 1.044) | 0.023 | 0.880 |
| GDF-15 (pg/mL)^a^ | -0.018 | (-0.069, 0.034) | -0.004 | 0.496 | -0.014 | (-0.068, 0.040) | -0.049 | 0.610 | -0.018 | (-0.073, 0.037) | -0.065 | 0.515 |
| sCD163 (pg/mL)^a^ | 0.002 | (-0.054, 0.058) | -0.007 | 0.944 | -0.013 | (-0.069, 0.042) | 0.053 | 0.642 | -0.013 | (-0.069, 0.043) | 0.065 | 0.643 |
| Leptin (pg/mL)^a^ | 0.043 | (-0.116, 0.201) | -0.005 | 0.597 | 0.018 | (-0.133, 0.169) | 0.141 | 0.813 | -0.010 | (-0.117, 0.097) | 0.573 | 0.856 |
| IL-6 (pg/mL) | 0.027 | (-0.276, 0.329) | -0.007 | 0.860 | 0.062 | (-0.236, 0.360) | 0.071 | 0.682 | 0.053 | (-0.245, 0.352) | 0.079 | 0.725 |
| IL-17A (pg/mL) | -0.240 | (-0.619, 0.138) | 0.004 | 0.211 | -0.285 | (-0.660, 0.091) | 0.070 | 0.136 | -0.263 | (-0.643, 0.117) | 0.060 | 0.174 |
| Models carried out as birthweight centile and cardiometabolic outcomes at 9-11 years. ^a^log10 transformed data was used. Abbreviations: CI Confidence interval; SBP Systolic blood pressure; DBP Diastolic blood pressure; 20-M SRT 20-metre shuttle run test; HOMA-IR Homeostatic Model Assessment for Insulin Resistance; TC Total cholesterol; LDL-C Low density lipoprotein cholesterol; HDL-C High density lipoprotein cholesterol; ICAM-1 Intracellular adhesion molecule 1; TNF-α Tumour necrosis factor alpha; GDF-15 Growth differentiation factor 15; sCD163 Soluble cluster of differentiation factor 163; IL Interleukin. Model 1: crude results; Model 2: adjusted for age at follow-up, study group allocation, sex, HP index, maternal age at delivery, maternal ethnicity, maternal early pregnancy BMI, gestational weight gain, maternal smoking in pregnancy; Model 3: adjusted for breastfeeding exposure, preteen BMI. | | | | | | | | | | | | |
