# Supplementary material for: The impact of macrosomia on cardiometabolic health in preteens: findings from the ROLO longitudinal birth cohort study
Source: Nutr Metab (Lond). 2023 Sep 4;20:37. doi: 10.1186/s12986-023-00759-8 (PMC10476328; doi:10.1186/s12986-023-00759-8)
Supplement: Supplementary file 5 — Additional file 5. Sensitivity analyses between macrosomia and preteen anthropometry and body composition [file 12986_2023_759_MOESM5_ESM.docx]

| Supplementary Table 5. Sensitivity analyses between macrosomia and preteen anthropometry and body composition. | | | | | | | | | | | | |
| --- | --- | --- | --- | --- | --- | --- | --- | --- | --- | --- | --- | --- |
|  | **Birthweight ≥4 kg** | | | | **Birthweight ≥4.5 kg** | | | | **Birthweight ≥90^th^ centile** | | | |
|  | B | 95% CI | R^2^ Adj | *p* | B | 95% CI | R^2^ Adj | *p* | B | 95% CI | R^2^ Adj | *p* |
| Weight (kg) | 0.743 | (-1.709, 3.194) | 0.353 | 0.550 | 2.195 | (-1.119, 5.510) | 0.360 | 0.192 | 1.361 | (-1.085, 3.806) | 0.358 | 0.273 |
| Weight z-score | 0.116 | (-0.209, 0.441) | 0.143 | 0.481 | 0.330 | (-0.108, 0.768) | 0.155 | 0.139 | 0.232 | (-0.091, 0.555) | 0.154 | 0.157 |
| Height (cm) | 1.002 | (-1.076, 3.081) | 0.419 | 0.341 | 2.583 | (-0.214, 5.381) | 0.430 | 0.070 | 2.237 | (0.188, 4.285) | 0.436 | 0.033 |
| Height z-score | 0.153 | (-0.180, 0.485) | 0.051 | 0.365 | 0.396 | (-0.052, 0.844) | 0.068 | 0.083 | 0.365 | (0.037, 0.692) | 0.081 | 0.029 |
| BMI (kg/m^2^) | 0.115 | (-0.807, 1.037) | 0.171 | 0.806 | 0.438 | (-0.814, 1.689) | 0.174 | 0.490 | 0.155 | (-0.767, 1.078) | 0.171 | 0.739 |
| BMI z-score | 0.075 | (-0.291, 0.441) | 0.094 | 0.687 | 0.218 | (-0.279, 0.715) | 0.099 | 0.387 | 0.090 | (-0.276, 0.457) | 0.095 | 0.626 |
| MUAC (cm) | 0.162 | (-0.809, 1.133) | 0.177 | 0.741 | 0.363 | (-0.957, 1.682) | 0.178 | 0.587 | 0.353 | (-0.618, 1.323) | 0.180 | 0.473 |
| WC (cm) | -0.492 | (-3.222, 2.238) | 0.208 | 0.722 | 0.675 | (-3.037, 4.387) | 0.208 | 0.720 | -0.213 | (-2.947, 2.522) | 0.207 | 0.878 |
| Sum of skinfolds (mm) | -0.531 | (-4.727, 3.664) | 0.210 | 0.802 | 1.417 | (-4.283, 7.117) | 0.212 | 0.623 | -0.272 | (-4.473, 3.929) | 0.210 | 0.898 |
| Subscapular/triceps ratio | -0.044 | (-0.117, 0.030) | 0.030 | 0.240 | -0.054 | (-0.153, 0.046) | 0.027 | 0.290 | -0.077 | (-0.150, -0.004) | 0.053 | 0.038 |
| Lean mass (kg) | 0.571 | (-0.545, 1.686) | 0.425 | 0.313 | 1.373 | (-0.130, 2.876) | 0.436 | 0.073 | 0.959 | (-0.149, 2.067) | 0.434 | 0.089 |
| Body fat (%) | -0.045 | (-2.413, 2.323) | 0.208 | 0.970 | 0.145 | (-3.075, 3.365) | 0.208 | 0.929 | 0.116 | (-2.254, 2.487) | 0.208 | 0.923 |
| Models carried out as macrosomia and anthropometry and body composition outcomes at 9-11 years. Abbreviations: CI Confidence interval; BMI Body mass index; MUAC Mid-upper arm circumference; WC Waist circumference. All models adjusted for age at follow-up, study group allocation, sex, HP index, maternal age at delivery, maternal ethnicity, maternal early pregnancy BMI, gestational weight gain, maternal smoking in pregnancy, breastfeeding exposure, preteen physical activity, sexual development. | | | | | | | | | | | | |
