# Supplementary material for: The impact of macrosomia on cardiometabolic health in preteens: findings from the ROLO longitudinal birth cohort study
Source: Nutr Metab (Lond). 2023 Sep 4;20:37. doi: 10.1186/s12986-023-00759-8 (PMC10476328; doi:10.1186/s12986-023-00759-8)
Supplement: Supplementary file 6 — Additional file 6. Sensitivity analyses between macrosomia and preteen cardiometabolic outcomes [file 12986_2023_759_MOESM6_ESM.docx]

| Supplementary Table 6. Sensitivity analyses between macrosomia and preteen cardiometabolic outcomes. | | | | | | | | | | | | |
| --- | --- | --- | --- | --- | --- | --- | --- | --- | --- | --- | --- | --- |
|  | **Birthweight ≥4 kg** | | | | **Birthweight ≥4.5 kg** | | | | **Birthweight ≥90^th^ centile** | | | |
|  | B | 95% CI | R^2^ Adj | *p* | B | 95% CI | R^2^ Adj | *p* | B | 95% CI | R^2^ Adj | *p* |
| SBP percentile | -0.266 | (-8.739, 8.206) | -0.077 | 0.950 | 1.799 | (-9.736, 13.333) | -0.076 | 0.758 | -0.328 | (-8.811, 8.155) | -0.077 | 0.939 |
| DBP percentile | -1.538 | (-10.326, 7.250) | -0.104 | 0.730 | -6.438 | (-18.356, 5.480) | -0.095 | 0.287 | -1.912 | (-10.709, 6.885) | -0.104 | 0.668 |
| Resting heart rate (bpm) | 1.884 | (-2.805, 6.573) | -0.084 | 0.428 | 0.601 | (-5.801, 7.003) | -0.089 | 0.853 | 0.399 | (-4.308, 5.106) | -0.089 | 0.867 |
| 20-M SRT score | 0.124 | (-0.310, 0.558) | 0.171 | 0.573 | 0.399 | (-0.188, 0.987) | 0.181 | 0.181 | 0.093 | (-0.342, 0.528) | 0.170 | 0.673 |
| HOMA-IR | -0.388 | (-1.608, 0.832) | -0.066 | 0.528 | 0.323 | (-1.341, 1.988) | -0.070 | 0.700 | -0.606 | (-1.823, 0.610) | -0.057 | 0.324 |
| TC (mmol/L) | -0.105 | (-0.436, 0.225) | -0.122 | 0.527 | -0.072 | (-0.523, 0.380) | -0.127 | 0.753 | -0.033 | (-0.366, 0.299) | -0.128 | 0.841 |
| Triglycerides (mmol/L) | -0.157 | (-0.453, 0.139) | -0.111 | 0.293 | 0.126 | (-0.280, 0.531) | -0.123 | 0.539 | -0.125 | (-0.422, 0.173) | -0.118 | 0.406 |
| LDL-C (mmol/L) | -0.006 | (-0.269, 0.258) | -0.099 | 0.967 | -0.149 | (-0.506, 0.208) | -0.088 | 0.407 | -0.003 | (-0.267, 0.260) | -0.099 | 0.980 |
| HDL-C (mmol/L) | -0.030 | (-0.211, 0.151) | -0.130 | 0.740 | 0.005 | (-0.242, 0.252) | -0.132 | 0.968 | 0.021 | (-0.160, 0.203) | -0.131 | 0.816 |
| C-reactive protein (mg/L) | -0.080 | (-0.673, 0.512) | -0.122 | 0.787 | -0.068 | (-0.875, 0.740) | -0.122 | 0.868 | -0.089 | (-0.683, 0.504) | -0.121 | 0.765 |
| C3 complement (g/L) | -0.094 | (-0.194, 0.007) | 0.213 | 0.067 | -0.073 | (-0.212, 0.066) | 0.187 | 0.298 | -0.066 | (-0.168, 0.036) | 0.193 | 0.202 |
| ICAM-1 (pg/mL)^a^ | -0.046 | (-0.125, 0.033) | -0.190 | 0.247 | -0.048 | (-0.156, 0.060) | -0.204 | 0.374 | -0.035 | (-0.115, 0.044) | -0.204 | 0.374 |
| TNF-α (pg/mL) | -0.817 | (-2.334, 0.700) | -0.141 | 0.284 | -0.202 | (-2.292, 1.888) | -0.168 | 0.846 | 0.040 | (-1.497, 1.577) | -0.169 | 0.958 |
| GDF-15 (pg/mL)^a^ | -0.044 | (-0.128, 0.040) | -0.204 | 0.298 | -0.004 | (-0.120, 0.112) | -0.231 | 0.945 | -0.022 | (-0.106, 0.063) | -0.225 | 0.609 |
| sCD163 (pg/mL)^a^ | -0.042 | (-0.130, 0.045) | -0.107 | 0.337 | -0.034 | (-0.154, 0.086) | -0.122 | 0.574 | -0.014 | (-0.102, 0.075) | -0.127 | 0.752 |
| Leptin (pg/mL)^a^ | -0.002 | (-0.160, 0.155) | 0.555 | 0.978 | -0.097 | (-0.309, 0.116) | 0.562 | 0.365 | -0.003 | (-0.160, 0.155) | 0.555 | 0.974 |
| IL-6 (pg/mL) | 0.020 | (-0.450, 0.489) | -0.078 | 0.933 | -0.128 | (-0.767, 0.511) | -0.074 | 0.689 | 0.042 | (-0.429, 0.512) | -0.077 | 0.860 |
| IL-17A (pg/mL) | -0.284 | (-0.890, 0.323) | -0.142 | 0.352 | -0.189 | (-1.020, 0.642) | -0.158 | 0.650 | -0.266 | (-0.873, 0.342) | -0.144 | 0.384 |
| Models carried out as macrosomia and cardiometabolic outcomes at 9-11 years. ^a^log10 transformed data was used. Abbreviations: CI Confidence interval; SBP Systolic blood pressure; DBP Diastolic blood pressure; 20-M SRT 20-metre shuttle run test; HOMA-IR Homeostatic Model Assessment for Insulin Resistance; TC Total cholesterol; LDL-C Low density lipoprotein cholesterol; HDL-C High density lipoprotein cholesterol; ICAM-1 Intracellular adhesion molecule 1; TNF-α Tumour necrosis factor alpha; GDF-15 Growth differentiation factor 15; sCD163 Soluble cluster of differentiation factor 163; IL Interleukin. All models adjusted for age at follow-up, study group allocation, sex, HP index, maternal age at delivery, maternal ethnicity, maternal early pregnancy BMI, gestational weight gain, maternal smoking in pregnancy, breastfeeding exposure, preteen BMI, preteen physical activity, sexual development. | | | | | | | | | | | | |
